# Mouth Self-examination for Prevention and Control of Oral Cavity Cancer

**DOI:** 10.31729/jnma.4910

**Published:** 2020-05

**Authors:** Gambhir Shrestha, Leison Maharjan

**Affiliations:** 1 Department of Cancer Prevention, Control, and Research, BP Koirala Memorial Cancer Hospital, Bharatpur, Chitwan, Nepal; 2 Department of Otolaryngology, Head & Neck Surgery, Patan Academy of Health Sciences, Lalitpur, Nepal

**Keywords:** *awareness*, *cancer*, *oral cancer*, *prevention*, *self-examination*

## Abstract

Oral cavity cancer is one of the most common preventable cancers in the world. The burden of the disease is high in South Asia. Therefore, public health strategies such as creating awareness and disease screening should be advocated for its prevention and early detection. Mouth self-examination serves both the purposes. It is easy to perform, non-invasive, and low-cost methods. It not only helps in the early detection of suspicious oral lesions but also helps people to quit their high-risk behaviors such as consumption of tobacco and alcohol.

## INTRODUCTION

Oral cavity cancer is one of the most common cancers in the world. The number of new cases of oral cavity cancer in the world is 354,864 in 2018 which is equivalent to an estimated risk of 2.0 per 100,000. Similarly, the number of deaths and estimated risk were 177,384 and 1.9 per 100,000 respectively.1 The trend of oral cavity cancer is rising in developing countries. Its prevalence is highest in South Asia.^1^

Oral cavity cancer is easily preventable and treatable if diagnosed early. However, most of the patients are not diagnosed until it reaches the late presentation and only half of those that develop the disease manage to survive after five years.^2^ It is a public health problem and without proper preventive strategies, its burden is likely to increase in the world. Consumption of tobacco including smokeless tobacco and alcohol are its major risk factors. Other risk factors are the consumption of betel quid and areca nuts, poor oral hygiene, Human Papilloma Virus infection, poor nutrition, weakened immune system, genetic and immunologic predisposition.^3^ In the majority of the cases, it is preceded by painless visible changes in the mouth known as precancerous lesions such as a whitish (leukoplakia) or reddish (erythroplakia) discoloration of the mucosa, an ulcer, or a swelling. If these changes are identified early, they can be treated with less invasive procedures with better outcomes.^4^ Yet patients seek medical attention only at an advanced stage.^5^ The reasons for the late presentation of the oral cancer are lack of information about the danger signs and lack of health-seeking behavior in case of premalignant lesions.^2^ Such delay in diagnosis leads to invasive treatment which leads to morbidity as well as a high economic burden to the patients.^6^

## SCENARIO OF ORAL CANCER IN NEPAL

Oral cancer is the sixth most common cancer in Nepal and also the sixth among the cancer deaths.^7^ The number of new cases of oral cavity cancer in Nepal was estimated at 1207 in 2018, which is equivalent to an estimated risk of 4.9 new cases per 100,000 men, while the number of deaths and the estimated risk was 845 and 3.4 per 100,000 respectively.^7^ In a study done in Eastern Nepal, 15% of the participants had oral potentially premalignant disorders.^8^ Most of the patients (41.8%) have not heard about oral cancer in a study done in Kathmandu Valley.^9^ This infers that many are unaware of the preventive measures as well against oral cancer. There needs to be a strong cancer registry in the country to register the cases of oral cavity cancer systematically and to devise preventive strategies.^10^ At present, there is no specific policy in Nepal for oral cancer screening. Most of our public health interventions for its prevention emphasize the risk reduction strategies such as cessation of tobacco and alcohol consumption only. However, few institutions such as B.P. Koirala Memorial Cancer Hospital periodically conducts screening camp in the community for oral cancer.

## MOUTH SELF-EXAMINATION AS A SCREENING TOOL

Known risk factors, long natural history, easy detection of precancerous lesions by oral examination make oral cavity cancer very suitable for population screening.^4^ Oral cavity cancer usually occurs at accessible sites, that lend themselves to early detection by visual inspection and palpation.^4^ Hence, mouth self-examination (MSE) is feasible to everyone as it is simple-to-perform, noninvasive, and low-cost method for early detection of oral precancerous lesions without the requirement of a healthcare professional.^4^ Also, MSE can lead to the self-perception of the need for early professional care. Hence it should be strongly advocated for the general population especially to the high-risk individuals.^11^ Moreover, it is also an effective way of increasing awareness of oral cancer and should be made a part of oral care behaviors.^12^ A quasi-experimental study conducted in Australia has concluded the importance of MSE in reducing morbidity and mortality in oral cancers.^13^ Similarly, the likelihood of an oral examination performed by a physician is greater when an individual practices MSE because the individual is more likely to perceive the need to seek professional care. As a result, MSE is likely to lead to an increase in the prevalence of the early diagnosis of disease resulting in the need for less invasive treatments.^14^ Another study also reported that the findings of MSE and health workers showed

72% concordance.^12^ Furthermore, MSE can also be very useful in treated oral cancer patients for evaluating disease status.^15^ Therefore, MSE can be the best strategy for oral cancer prevention along with the risk reduction strategy in low-resource countries like ours.

## MOUTH SELF-EXAMINATION AS A PART OF HEALTH EDUCATION

Health education is a very important intervention for health promotion and prevention of oral cavity cancer.^16^ Health education on MSE helps in early diagnosis and reduces the time duration between the detection of oral lesions and treatment. Health education programs are very effective in motivating people, especially high-risk individuals, which could result in regular MSE.^12^ Self-examination requires only a mirror and good lighting. But individuals may not be familiar with the normal appearance of their oral cavity and may not recognize the abnormal changes. Hence, health education on MSE is essential which unfortunately is still not being advocated in developing countries where the incidence of oral cavity cancer is quite high.^16^ Awareness programs on MSE is an important intervention to decrease the morbidity and mortality of oral cancer. Additionally, it also helps in prevention by quitting the high-risk behaviors among the public such as consumption of tobacco and alcohol.

In a study conducted in India, Sankaranarayan et al. found a significant reduction in mortality from oral cancer among individuals at risk when the examination was carried out by trained health professionals.^17^ Primary health care workers should be trained to screen high-risk patients for oral cancer regularly in their centers and to educate people on how to conduct MSE and report any abnormal lesions.

## WAY FORWARD

MSE is a cost-effective public health intervention. Not only it helps to raise awareness of oral cavity cancer, but it also leads to its early detection and reduces morbidity and mortality. Furthermore, it helps people to quit their high-risk behaviors such as consumption of tobacco and alcohol.

## Conflicts of Interest:

**None.**
